# Motorway Bottleneck Probability Estimation in Connected Vehicles Environment Using Speed Transition Matrices

**DOI:** 10.3390/s22072807

**Published:** 2022-04-06

**Authors:** Leo Tišljarić, Filip Vrbanić, Edouard Ivanjko, Tonči Carić

**Affiliations:** Faculty of Transport and Traffic Sciences, University of Zagreb, 10000 Zagreb, Croatia; ltisljaric@fpz.unizg.hr (L.T.); fvrbanic@fpz.unizg.hr (F.V.); tcaric@fpz.unizg.hr (T.C.)

**Keywords:** motorway bottleneck, connected vehicles, bottleneck detection, bottleneck probability, speed transition matrix, fuzzy-based bottleneck probability, traffic simulation

## Abstract

Increased development of the urban areas leads to intensive transport service demand, especially on urban motorways. To increase traffic flow and reduce congestion, motorway traffic bottlenecks caused by high traffic demand need to be efficiently resolved using Intelligent Transport Systems services. Communication technology development that supports Connected Vehicles (CVs), which act as an active mobile sensor for collecting traffic data, provides an opportunity to harness the large datasets to develop novel methods regarding traffic bottlenecks detection. This paper presents a speed transition matrix based model for bottleneck probability estimation on motorways. The method is based on the computation of the speed at the vehicle transition point between consecutive motorway segments, which forms a traffic pattern that is represented using transition matrices. The main feature extracted from the traffic patterns was the center of mass, whose position is used as an input to the fuzzy-based system for bottleneck probability estimation. The proposed method is evaluated on four different simulated motorway traffic scenarios: (i) traffic collision site, (ii) short recurring bottleneck, (iii) long recurring bottleneck, and (iv) moving bottleneck. The method achieves comparable bottleneck detection results on every scenario, with a total accuracy of 92% on the validation dataset. The results indicate possible implementation of the method in the motorway traffic environment with a high CVs penetration rate using them as the sensory input data for the control systems based on the machine learning algorithms.

## 1. Introduction

Bottleneck detection on motorways is a known and investigated research topic owing to the increased development of urban areas and intense transport service increase due to globalization [1]. The result of bottleneck occurrence is increased travel time, decreased traffic safety, and increased pollution due to stop-and-go driving.

Connected Vehicles (CVs) have emerged recently due to the development of communication technologies and Intelligent Transport Systems (ITS) services. Recently, many researchers are covering the CVs topic with the research regarding collaborative machine learning [2], CVs in the mixed traffic flow environment [3], CVs security [4], and upcoming challenges [5].

The traffic bottleneck is a phenomenon that can occur in urban and motorway roads. According to Ref. [6], the most common reasons for the bottleneck activation are merging of the on-ramp vehicles, lane drop, intense braking, fixed speed limits, and traffic incidents. All these events can be classified as unexpected driver behaviour on the motorway. In this paper, the proposed method for bottleneck detection is focused on detecting the unexpected behaviour that could lead to bottleneck start or the events that occur when vehicles are already in the bottleneck state. The bottleneck detection is conducted by observing the vehicle transitions between consecutive motorway segments. The bottleneck probability will rise when events like sudden breaks, stopping of the vehicle, or intense accelerations are detected.

As discussed in [7], standard traffic flow models are not able to represent traffic flow in a way to detect traffic breakdowns manifested in bottleneck occurrences. The CV technology enables the data collection in real-time from every vehicle on the traffic segments. Those data can be incorporated into emerging traffic models to detect bottlenecks efficiently and more accurately. In the context of mentioned research possibilities, this paper proposes a novel method for the road traffic bottleneck detection on motorways using the emerging traffic data modelling technique Speed Transition Matrix (STM) discussed further in [8,9]. The method is based on the CV data collection on the observed motorway, focusing on traffic patterns that emerge between consecutive motorway segments. Traffic patterns are represented with the STMs, while the bottleneck probability occurrence is estimated by applying the Fuzzy Inference System (FIS) with the position of the traffic pattern as the input variables. The main advantage of the proposed method is the ability to detect bottlenecks regardless of the current state of the traffic flow, and bottleneck type (moving, recurrent, etc.).

In this context, the contributions of this paper are as follows:

- Proposed method for the spatiotemporal motorway traffic patterns extraction which includes STM-based model;
- Proposed bottleneck detection method based on computation of parameters extracted from the STM;
- Bottleneck detection results evaluated using different motorway traffic scenarios that include collision site, recurrent, and moving bottlenecks prove the effectiveness of the proposed method.

This paper is organized as follows. Section 2 presents the literature overview of the methods and parameters used for road traffic bottlenecks detection. In Section 3, the background, definitions, and concepts used in this paper are explained. Section 4 presents the methodology overview for the proposed motorway bottleneck detection method. In Section 5, the simulation scenarios and used parameters are explained. Section 6 presents the results of the proposed method with the validation on the simulated dataset. Section 7 presents the advantages and disadvantages of the proposed method usage with the possible application directions. In Section 8, the conclusion and future work directions are given.

## 2. Literature Review

### 2.1. Traffic Parameters

The first step in bottleneck detection is choosing the traffic parameter that will be used. Many researchers use traditional traffic parameters to extract the traffic congestion areas and identify the bottleneck. The most common traffic parameters used for traffic data analysis and the road traffic scenario creation are speed, traffic flow, and density [10]. The observed parameters are commonly modelled as profiles, which are represented with vectors $A\in R^{m}$, where $A=(a^{\left(1\right)},a^{\left(2\right)},\ldots ,a^{\left(m\right)})$ and $a^{\left(i\right)}$ represents the observed traffic parameter in time interval i∈m [11]. The main disadvantages of the vector representation are its inability to represent spatiotemporal relations between multiple road segments, and values of the observed parameters are aggregated into narrow time intervals, leading to large deviations. On the other hand, spatiotemporal traffic data are often represented with the traffic matrix $B\in R^{m\times n}$, called traffic image due to a grid-based representation, commonly visualized with heatmaps [12]. Here, a respective traffic parameter is represented using matrix $B=(b^{\left(11\right)},b^{\left(12\right)},\ldots ,b^{\left(mn\right)})$, where *m* represents time intervals and *n* motorway segments. Matrix data representation can represent spatiotemporal data but requires more computing power and advanced analysis techniques. Speed and density are the most common parameters used in the research for bottleneck detection. In [1], authors propose computation of the congestion index based on the headway and density. Then, the bottleneck is identified by comparing the changes in the index in the spatial and temporal domain. In [13], authors use a speed threshold to detect congestion that is defined as n% of the average vehicle speeds on the observed road segment, where *n* varies between 10 and 90.

Transition models can be seen in 90 papers within Daganzo’s research [14]. The author presented a cell transmission model with a density as the main parameter for traffic state estimation. The main goal of the transition cell model was to observe the traffic parameter at the transition between two consecutive cells. The parameter difference due to the transitioning is then used as a measure for the representation of the motorway traffic flow.

In this paper, the speed transition model is proposed by using the STMs. STM does not suffer from data loss due to averaging the values into narrow intervals because speed data are not averaged in such a way. Average values of the speed at single road segments are computed using all collected speed data on the observed road segments. Thus, there are two challenges related to classic averaging that need to be addressed: (i) averaging thousands of scalar values often results in wide confidence ranges, and (ii) values represent the speed only on one road segment, with no data related to the interactions with the consecutive road segment. The STM averages the speed values not on one road segment, but on the transition point between two consecutive segments, which includes pair of two speeds that show the interaction between two adjacent road segments. By using the Center of Mass (CoM) measure, pairs of origin and destination speed values are averaged using the harmonic mean. Thus, speed for one vehicle is computed using harmonic mean, on origin and destination road segments for one transition, and placed into the STM. With this procedure, we weighted the speed average with the CoM, and spatiotemporal relations of the transition were captured. Thus, the change of the traffic pattern position in the matrix is observed during the transition, which provides a more visually interpretable way of representing and detecting the bottlenecks.

### 2.2. Bottleneck Detection

The authors of [15] analyzed existing bottleneck modelling techniques on the motorways. They concluded that models using fundamental traffic diagrams do not adequately describe the bottleneck phenomenon due to the decreased traffic flow and speed reduction observed in downstream traffic flows. Consequentially, there is a need to develop novel modelling methods that include interactions between consecutive road segments on motorways.

In [13], the authors proposed congestion bottleneck definition using computed congestion level cost and contagion cost related to congestion propagation to other road segments. The authors of [16] analyzed different urban network topologies and concluded that a strong community structure could improve the network performance to resist the propagation of the bottlenecks. The bottleneck was modelled using the cell transmission model to propagate the congestion through the downstream traffic flow from one road segment to another. In [17], the authors used the three-phase traffic theory to identify bottlenecks that started due to the high on-ramp inflow. The bottleneck is identified in the phase when vehicles are transitioning between free-flow and synchronized flow states. The authors distinguished two types of congestion patterns: synchronized flow patterns and general congested patterns. The authors of [18] analyzed empirical random phase transitions when vehicles were transitioning between the free traffic flow and synchronized traffic flow. The study showed that those transitions can occur randomly and can be used for bottleneck detection and analysis. In [7], the authors proposed a method for predicting moving bottlenecks by using probe vehicles data. The method is based on recognizing the phase transitions between the free flow and synchronized flow defined in Kerner’s three-phase traffic flow model to detect the bottleneck.

The main limitation of mentioned motorway bottleneck detection approaches is the inability to detect an already existing bottleneck on the observed road segments. The flexibility of the proposed STM-based approach in this paper fills the gap, and it can detect several different bottleneck scenarios, including an already existing bottleneck, moving bottleneck, and recurrent bottleneck. The second limitation of the observed approaches relates to the flexibility of the methods to consider a bottleneck’s length and duration. With the usage of the STM-based approach, this is achievable with counting the motorway segments affected by a bottleneck.

## 3. Background

### 3.1. Motorway Network Elements and Bottleneck Definitions

**Definition** **1.** *Motorway road network: Formally, road networks are represented as directed graphs G=(V,E), where E is a set of edges representing road network segments, and the intersections or connections between edges are represented with the set of vertices V. In the defined network, every edge $e^{\left(i\right)}\in E$ has a start vertex $v^{\left(i\right)}$ and end vertex $v^{\left(i+1\right)}$.*

**Definition** **2.** *Spatial transition: Movement of one vehicle between two edges $e^{\left(i\right)}$ and $e^{\left(i+1\right)}$ in time interval Δt is defined as a transition. There are two types of edges in the transition: origin edge $e^{\left(i\right)}$ and destination edge $e^{\left(i+1\right)}$.*

**Definition** **3.** *Speed transition: Vehicle’s travel throughout a transition in interval Δt, where two speeds are measured to compute the speed transition: speed on the origin edge $v_{o}$ and speed on the destination edge $v_{d}$. Speeds are computed as a harmonic mean speed to emphasize the importance of the smaller values to capture low-speed values.*

**Definition** **4.** *Road traffic states: In this paper, three road traffic states categories are defined based on [8,19]: (i) free-flow, (ii) unstable traffic flow, and (iii) congested traffic flow. Free-flow is defined as traffic conditions with no interactions between vehicles due to low traffic density. Thus, speeds at spatial transitions are close to speed limits. The unstable traffic flow is represented by the traffic conditions with some interaction between vehicles due to increased traffic density. The speeds at spatial transitions are approximately 50–80% of the defined speed limit. The congested traffic flow is characterized by traffic jams with speeds at spatial transitions close to zero.*

**Definition** **5.** *Motorway bottleneck: Normal traffic conditions can be described with traffic flow $q_{in}\approx q_{out}$, where $q_{in}$ stands for upstream traffic flow and $q_{out}$ represents downstream traffic flow on the observed part of the motorway. The bottleneck is manifested by decreasing the downstream traffic flow due to the congestion. In this paper, three distinct traffic situations are defined as bottlenecks: (i) sudden breaks, as the start of a bottleneck where vehicles are approaching the congested area, (ii) heavy congested area, where vehicles are slowed down or not moving, and (iii) sudden acceleration area, where vehicles are leaving the congested area. With those events, three bottleneck scenarios can be described: the start of the bottleneck, vehicles in the bottleneck, and the bottleneck clearance. In this paper, the bottlenecks are described using the proposed bottleneck probability metric $p_{b}$. The $p_{b}$ will show the probability of bottleneck occurrence in the observed motorway segments with the values [0,1]. Here, value of 0 represent the traffic state with no interactions between vehicles, while the value of 1 represents the occurrence of one of the mentioned traffic bottleneck scenarios. Thus, $p_{b}$ will not show the distinction between three scenarios, but show the bottleneck probability if the scenario occurs.*

### 3.2. Speed Transition Matrix

**Definition** **6.** *The STM captures the vehicle’s speed at the spatial transition to represent the speed probability change, and therefore it represents the speed probability distribution at one spatial transition in Δt. The harmonic speed is chosen as the traffic parameter regarding its property of favouring the lower values during the aggregation compared to average speed. This property enables recognition of potential bottleneck generation even with the low amount of measured vehicle speeds with high deviations. Measure vehicle speed is represented relative to the speed limit on the motorway road segments that are observed.*

*The STM can be represented with the expression:*

(1) $$X^{\left(ij\right)}\left(\Delta t\right)=\;\left(\begin{array}{cccc} p^{\left(11\right)} & p^{\left(12\right)} & \cdots & p^{\left(1n\right)}\\ p^{\left(21\right)} & \ddots & \; & \vdots \\ \vdots & \; & \ddots & \vdots \\ p^{\left(m1\right)} & \cdots & \cdots & p^{\left(mn\right)} \end{array}\right),$$

*where $X^{\left(ij\right)}\left(\Delta t\right)$ represents the STM between edges $e^{\left(i\right)}$ and $e^{\left(i+1\right)}$ observed in the time interval Δt. Indexes m and n represent relative traffic speeds with discrete values of 5% relative to the 100% as the maximal possible relative speed, which represent the respective speed limit. This process resulted in matrix dimensions of 20 × 20.*

**Figure 1:**
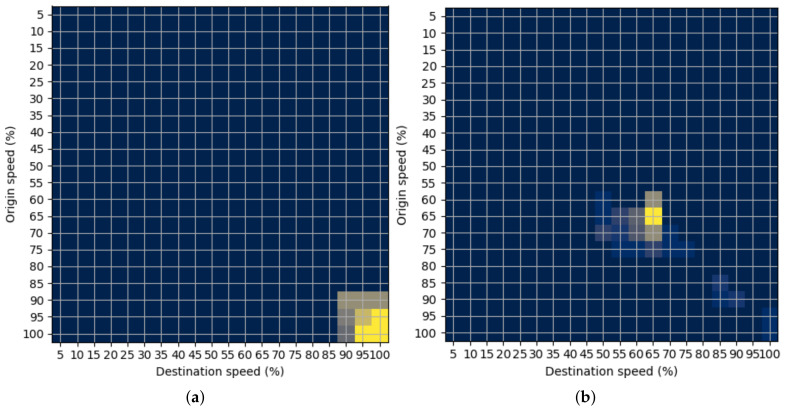

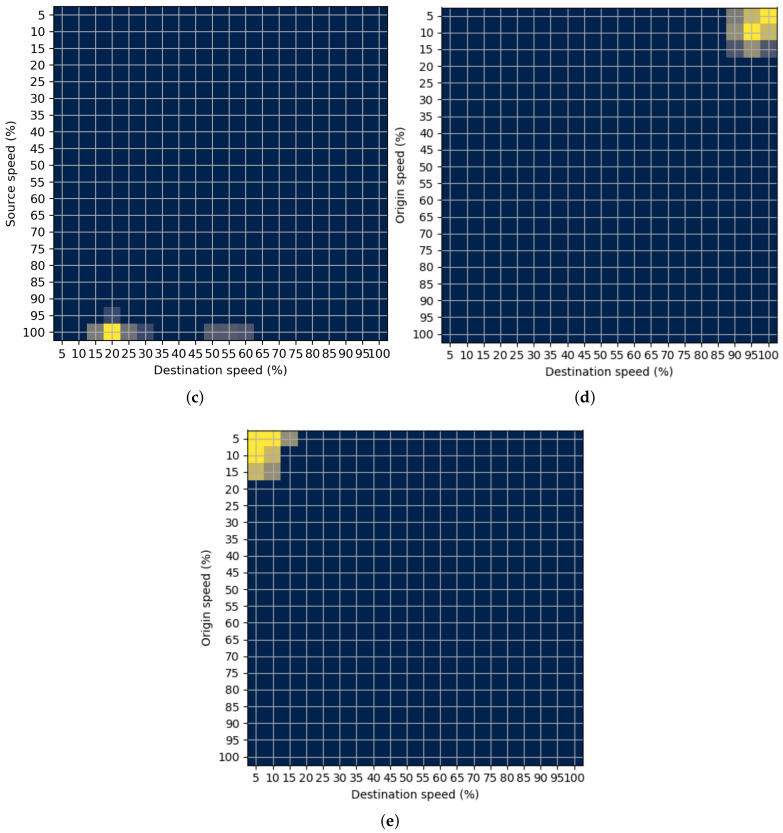
Examples of the characteristic STMs. (**a**) Free flow; (**b**) Unstable flow; (**c**) Bottleneck start; (**d**) Bottleneck end; (**e**) Heavy congestion.

There are five examples of the characteristic STMs on the motorway visually represented in Figure 1. Colors in the figures represent the speed change probability at the observed transition between two consecutive road segments labelled as origin and destination segment. Dark colors represent low probability, while light colors represent high probability of speed transition. The first example in Figure 1a shows that vehicles on the observed transitions had both origin and destination speeds, close to 100% of the speed limit. Thus, the traffic state can be defined as free-flow with no congestion. The second example in Figure 1a shows the more unstable traffic state because vehicles have speeds close to 60% of the speed limit. This event can indicate the start of the congestion, but it cannot represent the start of the bottleneck regarding the relatively high speeds. Figure 1b,c represent the start and the end of the bottleneck, respectively. The bottleneck’s beginning is characterized by the transitions from high-speed values to low values because vehicles are transitioning from free-flow (or unstable flow) to congested flow. On the other hand, the bottleneck’s end is characterized by the opposite event. Here, vehicles are transitioning from congested traffic flow to free flow. The last example in Figure 1e shows the congested traffic state that occurs “inside” the bottleneck and is characterized by very low speeds on one transition’s origin and destination segments.

From the examples in Figure 1 it can be observed that the position of the observed traffic pattern, represented by the STM, has a crucial role in determining the traffic state on the observed motorway traffic segments. This is why CoM is chosen as a method for extracting the traffic state in this paper. The CoM estimation method is covered in more detail in the next section.

## 4. Methodology

**Figure 2:**
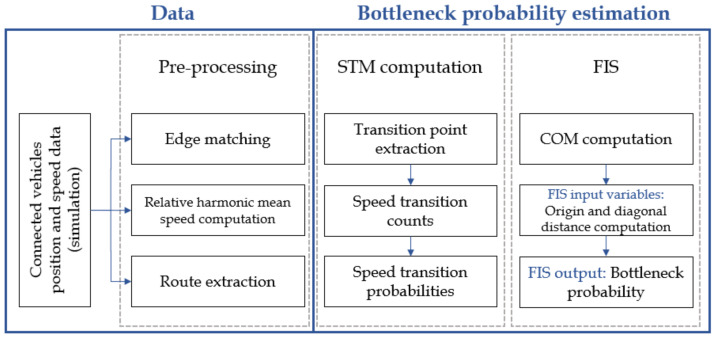
Overview of the methodology for the bottleneck probability estimation.

This paper aims to propose a methodology for motorway bottleneck probability estimation using traffic patterns extracted from the STMs. The overview of the methodology is presented in Figure 2 with three main steps. The first step is data preprocessing, which includes simulated vehicle position data preparation as input for STM computation. For every vehicle, its route is extracted with corresponding relative harmonic speed values, and it is matched to the corresponding edge. The second step includes the computation of the STMs, which represent the speed probability distribution for every vehicle travelling between two consecutive edges. Then, the last step includes FIS for bottleneck probability estimation with input variables computed as CoM distances from the origin and diagonal of the STM, respectively.

**Figure 3:**
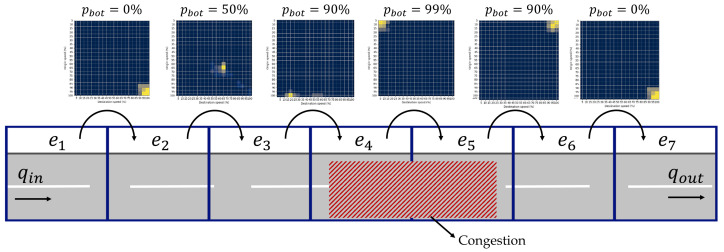
Overview of the proposed method for the bottleneck probability estimation.

The bottleneck probability estimation is further explained in depth with the example in Figure 3. This example shows a simple motorway divided into seven road segments labelled as edges $e^{\left(i\right)}$ where i∈1,2,…7. Every STM represents the traffic state on the transition between two edges. The input and the output traffic flows are labelled with $q_{in}$ and $q_{out}$. The traffic bottleneck begins at the edge $e^{\left(4\right)}$ and spans to $e^{\left(5\right)}$, which is caused by congestion. The proposed method is estimating the bottleneck probability for every edge based on the characteristic STMs explained in Section 3.2.

To amplify the importance of the location of the patterns, the method for the motorway bottleneck detection consists of three parts: (i) estimation of the CoM for every traffic pattern represented with STM, (ii) computation of the distance between CoM and the diagonal of the STM, and (iii) computation of the distance between CoM and the origin of the STM. The next subsections explain each of the parts in detail.

### 4.1. Center of Mass Estimation

To successfully detect the motorway bottleneck caused by the congestion using the STM, the position of the traffic pattern represented by the STM is crucial. The position of the traffic pattern is used as the main feature in estimating the traffic state on the motorway. In this paper, the CoM, based on the computation of the expected value, adopted from [20], is chosen as a method for detecting the position of the traffic pattern. To compute CoMs, expected values of the coordinates (origin and destination speed) are computed using Equations (2) and (3):

(2) $$c_{x}=\sum_{j=1}^{n}p_{x}\left(x^{\left(j\right)}\right)\cdot j,$$

(3) $$c_{y}=\sum_{i=1}^{m}p_{y}\left(y^{\left(i\right)}\right)\cdot i,$$

where $c_{x}$ and $c_{y}$ represent coordinates of the CoM and $p_{x}\left(x^{\left(j\right)}\right)$ and $p_{y}\left(y^{\left(i\right)}\right)$ represent the marginal distributions for the coordinates. Further explanation of the method can be found in our previous works [8,21].

### 4.2. Fuzzy Inference System

**Figure 4:**
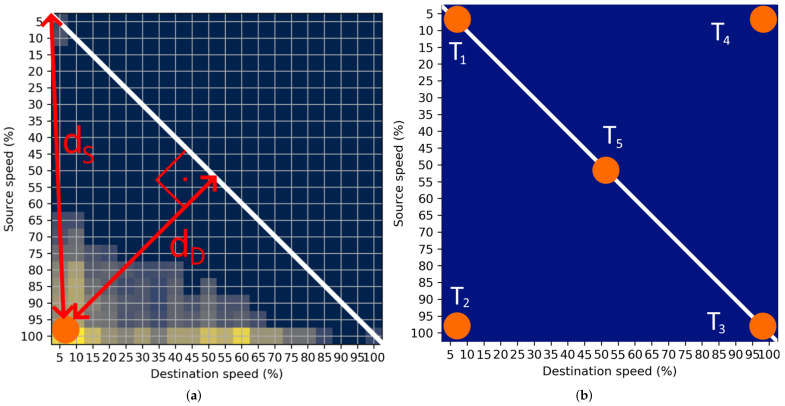
Method for FIS input variables computation. (**a**) Example of the $d_{S}$ and $d_{D}$ computation; (**b**) CoM positions of characteristic STMs.

When the CoM is computed, appropriate features must be extracted to quantify the bottleneck probability. In this paper, two features are extracted from the computed CoM: (i) distance from the origin of the STM labelled as $d_{S}$, and (ii) distance from the diagonal of the STM labelled as $d_{D}$. Two features are shown in the example in Figure 4a.

The first feature, $d_{S}$, is important for estimating the traffic state on the observed transition. Let us examine some extreme points represented in the Figure 4b. At point $T_{1}$, $d_{S}$ is small. Then, the STM represents the transition of vehicles that had very low speed during the transition in the origin and destination segments, which can be declared as heavy congestion. On the other hand, at point $T_{3}$, $d_{S}$ has its largest value. Then, represented speeds will be very high (relative to the speed limit) in the origin and destination segments, which can be declared as free-flow conditions. At point $T_{5}$, feature $d_{S}$ is at 50% of the maximal value. This event can be declared as unstable traffic flow, as described in Figure 1b. It can be concluded that if the CoM is at the diagonal of the STM, $d_{S}$ can be effectively used for the estimation of the traffic state.

However, the second feature must be introduced to represent other possible positions of the CoMs, for example, at points $T_{2}$ and $T_{4}$. At these points, feature $d_{S}$ is at 70% of its maximal value, leading to a false conclusion that the traffic state is close to free-flow. Additionally, the second feature $d_{D}$ is at its maximal value and gives crucial information about the traffic state. At point $T_{2}$, the start of the bottleneck can be observed because of transitions from very large origin speeds to very low destination speeds. On the other hand, at $T_{4}$, clearance of the bottleneck can be observed as the origin speeds of the transition are low, and destination speeds are very high.

As features $d_{S}$ and $d_{D}$ can be represented as linguistic variables, an appropriately set FIS is used to detect the probability of the bottleneck occurrence. Fuzzy rules set for the bottleneck probability estimation is presented in Table 1. The rules were created using expert knowledge about the bottleneck definition extracted from [8,19]. An important consideration for rule setting was to address the behaviour of the two input parameters $d_{d}$ and $d_{s}$ with the corresponding correlations.

**Figure 5:**
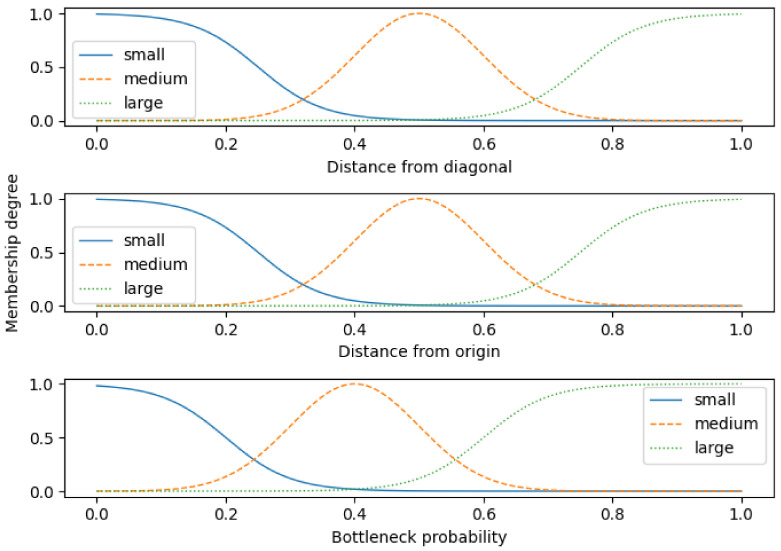
Initial FIS setup for the bottleneck probability estimation.

Figure 5 represents the setup of the proposed FIS for bottleneck detection. It consists of two input variables $d_{D}$ and $d_{S}$ with corresponding output $p_{b}$ that represents bottleneck probability. All variables are represented with range [0,1], relative to their maximal values. The maximal value of the $d_{S}$ is the length of the STM diagonal that can be computed as $20\sqrt{2}$, while the maximal value of $d_{D}$ can be computed as $d_{S}/2$.

Every variable is represented using three linguistic expressions, “small”, “medium”, and “large”, representing the possible states of the variables. The term “small” is represented using the Z-type membership function, “medium” is represented by Gaussian function, and “large” is represented by S-type membership function. The bottleneck probability variable has an offset of 0.1 to the left to allow the system to be more sensitive to speed changes.

## 5. Simulation of Motorway Traffic

### 5.1. Simulation Setup

The framework for motorway bottleneck estimation was made in SUMO traffic simulator [22] coupled with Python programming language script via TraCI interface [23]. The simulated motorway model mainstream is 8 km long and segmented into 500 m road segments for testing and 50 m length road segments for validation, totalling 16 and 160 road segments, respectively. Different road segments’ lengths are chosen to test the method’s ability to adopt to different scenarios of collecting traffic data, regardless of the segment’s length. The motorway model has one on-ramp and one off-ramp. The simulation lasts for two hours of traffic simulation, totalling 24 of 5 min intervals. Data for each vehicle and road segment is collected cumulatively every second for each 5 min interval. Harmonic mean speed and density are computed for each road segment for every interval.

### 5.2. Traffic Scenarios

**Figure 6:**
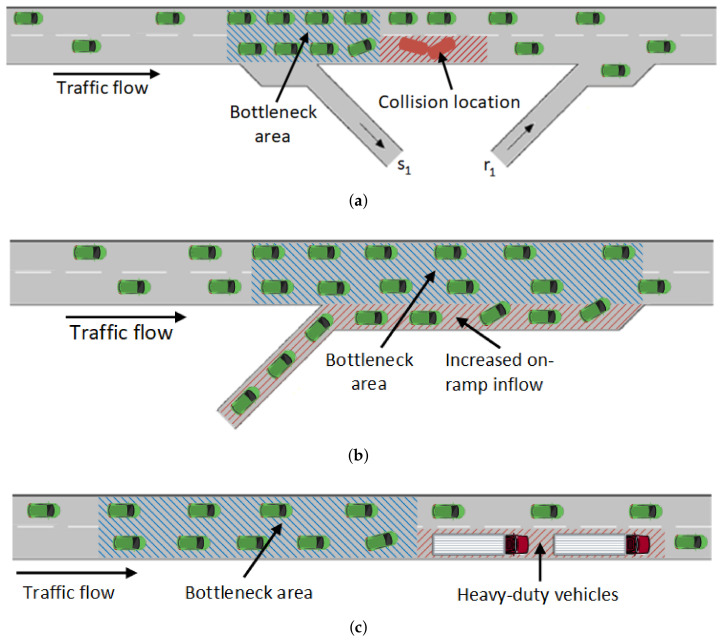
Analyzed simulation scenarios. (**a**) Collision scenario; (**b**) Increased on-ramp inflow scenario; (**c**) Heavy-duty vehicles scenario.

To evaluate the proposed method, four traffic scenarios for simulation were created as shown in Figure 6. It has to be noted that two cases were created using the increased on-ramp inflow scenario given in Figure 6b. The first scenario (Figure 6a) simulates the collision in one lane on the motorway. The collision starts at the 1-hour mark and lasts for 30 min. The collision is simulated in the right-most lane. The mainstream flow was set to 2400 veh/h with the increase to 2800 veh/h between the 15th and 55th minute to simulate the peak inflow of vehicles. The on-ramp flow was set to 600 veh/h with the increase to 1100 veh/h between the 15th and 55th minute to simulate the peak inflow of vehicles to the mainstream flow. The goal of this scenario is to test the method’s ability to detect a bottleneck when a traffic incident occurs.

The second and third scenarios (Figure 6b) were created to simulate the increased inflow of on-ramp vehicles. The second scenario had the same mainstream flow as the first scenario, while the on-ramp flow was set to 600 veh/h and was increased to 1200 veh/h between the 15th and 55th minute to simulate the increased peak inflow of vehicles to the mainstream flow. The third scenario had the same mainstream flow as the first scenario, while the on-ramp flow was set to 600 veh/h and was increased to 1400 veh/h between the 15th and 55th minute to simulate a very high inflow of vehicles to the mainstream flow. The second and third scenarios show the method’s ability to detect recurrent bottlenecks due to daily commuters or similar recurrent events.

The fourth scenario simulates a high inflow of Heavy-Duty Vehicles (HDVs). The inflow of vehicles on the mainstream and on-ramp was set to be the same as for the first scenario. With this scenario, the proposed method is tested against the detection of moving bottleneck caused by slow HDVs.

## 6. Results

The proposed bottleneck probability estimation method was evaluated on a synthetic dataset extracted from the SUMO traffic simulation framework. The method was evaluated on four different possible motorway congestion scenarios that originate from different sources of congestion, namely: traffic accident, short recurrent congestion due to high on-ramp inflow, long recurrent congestion due to high on-ramp inflow, and moving bottleneck originating from a substantial amount of slow HDVs on the motorway.

### 6.1. Data

The most important parameters extracted from the simulation are vehicle speed, traffic density on the observed link, and the location of the vehicles on the motorway. Table 2 presents recorded parameters extracted from the simulation.

Two types of datasets were extracted from the simulation: edge data and vehicle routes data. Traffic parameters for every observed motorway road segment in the edges dataset are collected in 5 min time intervals. For every edge, harmonic vehicle speed and density is computed. The density is computed by using the number of vehicles by an edge. STMs cannot be computed from this dataset because there are no vehicle routes information. Therefore, this dataset is used for testing and validation of the method. The second dataset presents vehicles’ routes recorded and collected in 5 min time intervals. The vehicle route is extracted for every vehicle on the observed motorway containing speed and location parameters. From this dataset, STMs are computed by counting the speed transitions between consecutive motorway segments.

### 6.2. Bottleneck Probability Estimation

**Figure 7:**
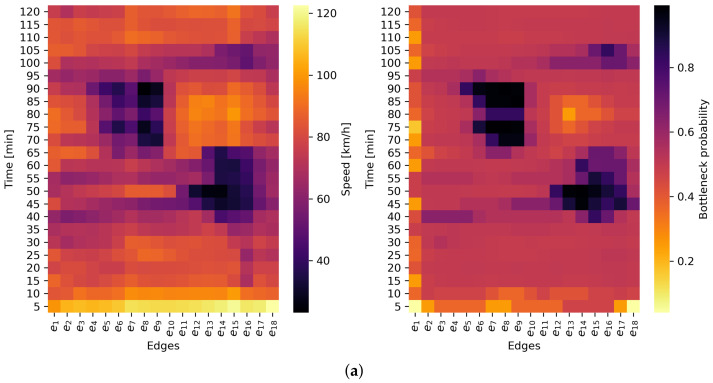

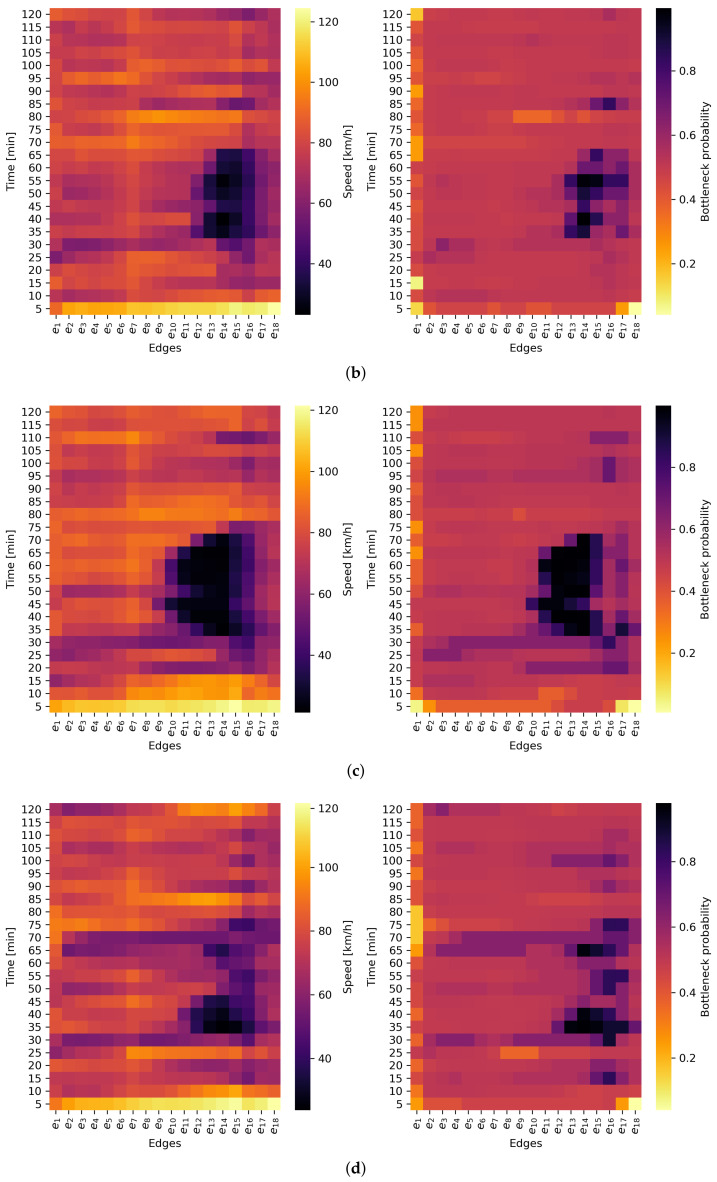
Results of the comparison between absolute harmonic speed measurements (**left column**) and proposed bottleneck probability estimation method (**right column**). (**a**) Scenario 1—collision site; (**b**) Scenario 2—recurring short bottleneck; (**c**) Scenario 3—recurring long bottleneck; (**d**) Scenario 4—moving bottleneck.

The results of the bottleneck probability estimation were compared to measured average mean speed values across every scenario. The comparison was made in the spatiotemporal domain presented in Figure 7. From Figure 7a–d, every example consists of two images: the image on the left shows the harmonic mean speed values, and the image on the right shows values of computed bottleneck probability. In the temporal domain, every cell represents one 5 min time interval, and spatially, the motorway is divided into 500 m edges.

In the first example in Figure 7a, the bottleneck is caused by two traffic events. The first is high on-ramp inflow which started at the 15th minute and lasted until the 55th minute. The second event is a traffic accident that occurred at the 60th minute. The left image shows that the speed drops at the 15th and 65th minutes and two bottlenecks occurred at the 45th and 65th minutes. The proposed method was able to detect both congested situations that resulted in bottleneck formation.

The second example in Figure 7b presents the bottleneck due to the short recurrent congestion caused by an increased inflow of vehicles to mainstream flow from an on-ramp. The bottleneck starts to form at the $e^{\left(10\right)}$ to $e^{\left(13\right)}$ at the 35th minute and lasts until the 65th minute of the simulation time. The speed values indicate the speed decrements even between the 10th and 15th minutes, which could be misleading for the control algorithm at the motorway. On the other hand, $p_{b}$ values start to increase between the 30th and 35th minutes, which provide a more accurate time for the response of the motorway control algorithm.

The third example in Figure 7c presents the bottleneck caused by the very high recurrent congestion due to an increased inflow of vehicles to mainstream flow from an on-ramp. The bottleneck spans from the $e^{\left(9\right)}$ to the $e^{\left(17\right)}$ within the time interval from the 35th to the 70th minute. The $p_{b}$ values start to increase at the 30th minute of the simulation, especially at the $e^{\left(16\right)}$, at which point the start of the bottleneck formation can be identified.

The fourth example in Figure 7d presents the moving bottleneck caused by the slow HDVs on the motorway. In the observed scenario, a very large number of HDVs entered the motorway, which resulted in a moving bottleneck that started at the 65th simulation minute. The consequences of this type of bottleneck are reflected with the increased $p_{b}$ values on a large number of edges, especially at the 70th minute. The speed values confirm the claim with the speed decrease in the whole motorway after the entrance of the HDVs.

### 6.3. Validation

The first step in the validation process was to create the ground truth dataset to compare it with the proposed method. The main goal was to estimate the bottleneck probability using common traffic parameters like speed and density and find a corresponding threshold for the bottleneck detection. For the validation process, the motorway scenario representing the collision site is chosen (Figure 7a).

Critical density $\rho_{c}$ was estimated according to [19], where the value of 28 veh/km/lane was reported as a critical value. When the vehicles on the motorway operate under $\rho_{c}$ condition, the traffic flow is maximal. If the current density is above the critical one $\rho >\rho_{c}$, congestion occurs, which consequentially leads to a bottleneck start. Another used traffic parameter was the critical speed $v_{c}$. Authors in [13,19,24,25,26], reports critical speed values from 50–60% of the free flow speed. In this paper, the value of 55% of the free flow speed is chosen as the critical speed on the motorway. According to the Highway Capacity Manual [19], at this speed, serious congestion with the lowest level of service is certain.

**Figure 8:**
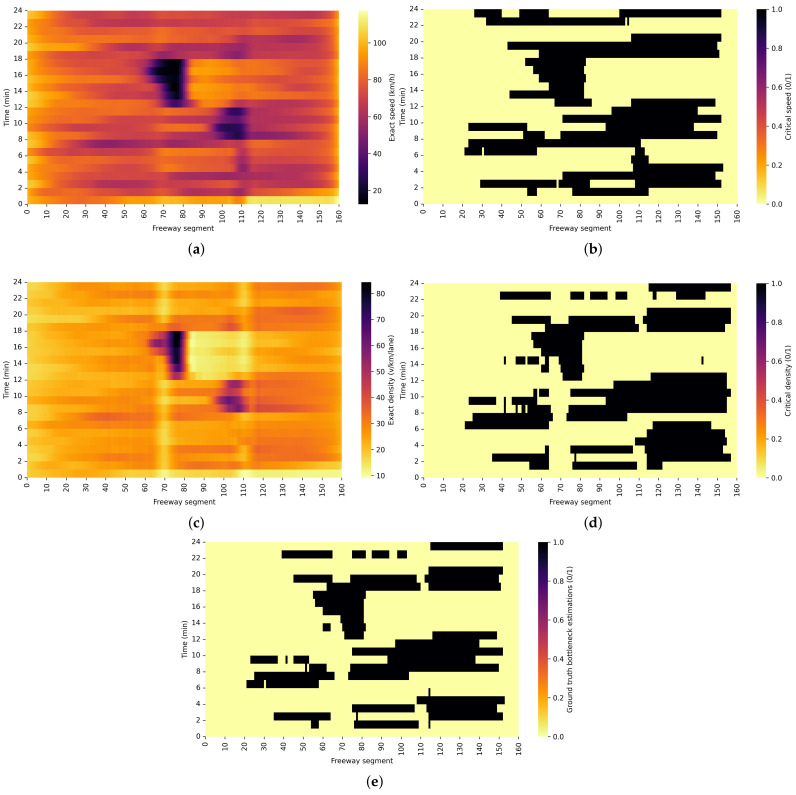
Ground truth data creation process for validation of the proposed method. (**a**) Exact values of the speed measurement; (**b**) Binary image where 1 represents critical speed; (**c**) Exact values of the density measurement; (**d**) Binary image where 1 represents critical density; (**e**) Intersection of critical speed and density values.

Speed and density were measured on all observed 50 m motorway segments for the creation of the ground truth dataset. The harmonic mean speed and density were collected for each road segment. The exact values of parameters are shown in Figure 8a,c for the observed time period of 125 min divided into 5 min intervals where the dark colour represents the low values of the speed and the high values of the density. The corresponding critical values are shown in Figure 8b and the binary image containing critical speed values can be represented with matrix $V\in R^{m\times n}$. The elements of the matrix $v^{\left(ij\right)}$ are binary values expressed as:

(4) $$V^{\left(ij\right)}=\left\{\begin{array}{c} 1,v^{\left(ij\right)}\leq v_{c}\\ 0,v^{\left(ij\right)}>v_{c} \end{array}\right.,$$

where *m* represents the number of time intervals, and *n* represents the number of motorway segments.

On the other hand, the binary image containing critical densities (Figure 8d) can be represented with matrix $D\in R^{m\times n}$, where elements of the matrix $\rho^{\left(ij\right)}$ are also binary values expressed as:

(5) $$D^{\left(ij\right)}=\left\{\begin{array}{c} 1,\rho^{\left(ij\right)}\geq \rho_{c}\\ 0,\rho^{\left(ij\right)}<\rho_{c} \end{array}\right..$$

Finally, Figure 8e represents the intersection of the critical values $P_{eval}=V\cap D=\left\{x:x\in V,x\in D\right\}$. The intersection of critical values of speed and density $P_{eval}$ represent the bottleneck occurrence on the observed motorways. There, variable *x* will contain values 0 if a bottleneck is not detected, and 1 if the a bottleneck is detected. The matrix $P_{eval}$ is further used as a ground truth data.

The next challenge was to adopt the results of the proposed method for the comparison with the ground truth data. As the result of a bottleneck probability estimation is a decimal number in the range [0,1]. The probability threshold for the bottleneck detection must be defined to discretize the values to 1 if the bottleneck was detected and 0 if not. Table 3 shows the result of the threshold estimation. Estimating the probability threshold was conducted by changing the threshold values α from 10% to 90% and reporting the precision, recall, and F1-score between ground truth data and discretized the proposed method. It can be observed that the threshold of 50% gives the best results with an accuracy score of 0.92. Formally, the result of the proposed method can be represented by the matrix $P_{prop}\in R^{m}\times n$ with the values expressed as:

(6) $$P_{prop}^{\left(ij\right)}=\left\{\begin{array}{c} 1,p_{bot}^{\left(ij\right)}\geq \alpha \\ 0,p_{bot}^{\left(ij\right)}<\alpha \end{array}\right.,$$

where $p_{bot}^{\left(ij\right)}$ represents the bottleneck probability and α represents defined threshold value for the discretization.

**Figure 9:**
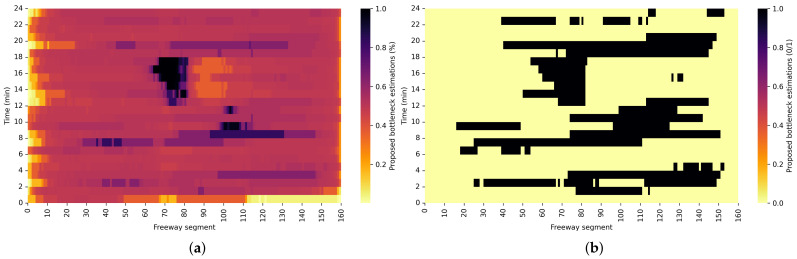
Proposed method for the bottleneck probability. (**a**) Estimated bottleneck probability exact values; (**b**) Binary image where 1 represent bottleneck.

With the defined threshold, any value of the bottleneck probability greater than 50% will be considered a detected bottleneck and represented with the value of 1. Bottleneck probabilities for the observed validation scenario are presented in Figure 9a with the corresponding binary values in Figure 9b.

## 7. Discussion

This paper presents a novel motorway bottleneck detection approach based on the STM-based traffic data model. This section aims to describe the method’s application possibilities and address the disadvantages and considerations for using the method. The emphasis of the proposed method is given on the impact of future academic research related to traffic control strategies on urban motorways and the usage of artificial intelligence methods.

Alongside the advantages and presented application of the proposed method, some considerations must be addressed. The method is evaluated on a dataset produced with simulated CVs on the motorway. Currently, all vehicles are not connected and integrated into one communication system. Accordingly, the method could be evaluated on a dataset, which contains mixed traffic flow (containing CVs and not connected vehicles) to be applicable to the current motorway scenarios. On the other hand, speed is used as the only traffic parameter for bottleneck detection. Introduction of several more transition matrices containing parameters like density or traffic flow could increase the accuracy and reliability of the method’s result in more versatile motorway traffic scenarios. Final consideration when using STMs is the trade-of between the time interval of data collection for one matrix Δt and the traffic pattern accuracy, represented by the STM. The Δt depends on the type of the research that is conducted. For macroscopic considerations, like traffic state estimation or finding the anomalies in large datasets, interval can be set to more wide range, with intervals of one hour or larger. In this case, many different traffic patterns are captured by the STM, but the most dominant can be extracted. On the other hand, if the research is related to micro scale like traffic control on highway or intersections, Δt should be set to a minutes scale. In this paper, the interval was set to 5 min, which was used for STM data collection, and is appropriate for bottleneck detection on motorways.

### 7.1. Features for Bottleneck Probability Estimation

The STM represents the speed change in one spatial transition observed on the motorway. In the process of recording the speed transition, a spatiotemporal correlation between consecutive road segments in the form of the speed pair is captured. In this way, the speed transition will show the weighted average of the speed pairs rather than a simple averaging technique. The proposed STM-based approach can be compared with the methods that rely on a combination of multiple parameters to get more correct values by combining two or more parameters or sensor values like Kalman filter or Hough transform.

Extracted attributes $d_{S}$ and $d_{D}$ represent the positional information for the pattern extracted from the STM with the additional information related to two important research topics in the field of ITS, traffic state estimation and anomaly detection. The value of the $d_{S}$ represents the traffic state with values in the range [0,1], where 0 represents congested traffic state, and 1 represents the free-flow traffic. On the other hand, $d_{D}$ addresses the anomaly measure in the traffic data with the values range in [0,1] where the 0 represents normal traffic behaviour with $d_{S}$ as an only important attribute, and 1 represents the anomaly in the observed traffic pattern. Combining those two attributes provides complete insight into the traffic state. It allows one to analyze the traffic behaviour with one or both features, depending on the considered use case.

### 7.2. Motorway Traffic Control Strategies

There are two most researched motorway traffic control systems, namely, Variable Speed Limit (VSL) [27] and Ramp Metering (RM) [28]. The goal of both control systems is to harmonize traffic flow on the main flow at the motorway to prevent bottleneck occurrence, reduce congestion, decrease travel time, and decrease pollution. VSL consists of traffic signs positioned along the motorway that show the current speed limit to the drivers encountering some traffic problem at the downstream traffic flow. On the other hand, RM consists of a traffic light at the motorway on-ramp and regulates the number of vehicles that can access the main traffic flow.

Recent studies [29,30] are proposing the usage of a virtual VSL suited for CVs that will receive speed limit information directly to the car dashboard. The STM-based traffic data modelling approach provides a more insightful data model as an input to both motorway traffic control systems. The bottleneck detection method proposed in this paper presents insights into the traffic state at the downstream traffic flow. It could provide actionable information to adapt the speed limits to harmonize traffic flow. The second important information extracted by the proposed bottleneck detection approach is the bottleneck length and duration propagation through spatial and temporal components. The propagation can be simply captured and implemented by counting the number of cells inflicted by the bottleneck in the spatial and temporal domain, respectively.

### 7.3. Reinforcement Learning Methods

The authors in [31] summarized different reinforcement learning methods to VSL on the motorways in mixed traffic flows that were used to improve the performance of the control system. The emphasis of research in [32,33,34] is using Connected Autonomous Vehicles (CAVs) as the mobile sensors that provide data for cooperative VSL, which is used as a speed control system on a motorway. The main goal was to use CAVs to maximize the mainstream traffic flow for reducing the delay time of vehicles by adjusting a motorway speed limits with the appliance of appropriate speed limits. Apart from using the CAVs as actuators, they can be used for state estimation when dealing with Q-learning [34]. By doing so, the classical traffic measurements such as speed, flow, and density can be replaced with the state estimation of bottleneck probability on a sequence of small segments on a motorway. Thus, using the bottleneck probability as an input to the Q-learning could potentially increase the performance of VSL and allow finer calibration of the VSL system and improve performance.

## 8. Conclusions

Emerging technologies like CVs present challenges for developing new methods for traffic data analysis, traffic state estimation, and control strategies. Thus, CVs can be mobile sensors and actuators. In this paper, the method for motorway traffic bottlenecks probability estimation is proposed. The method is based on the STM computation and extraction of the represented traffic parameter position as an input to the fuzzy-based algorithm. The result of the method was the bottleneck probability estimated on the simulated motorway traffic scenarios. The method showed comparable results on four different traffic scenarios including traffic incident, recurrent traffic, and the moving bottleneck, with a validation accuracy of over 92%. The proposed method can be used in an environment with high penetration rate of the CVs and as an input to the motorway traffic control algorithms.

Future work relates to the implementation of the STM-based bottleneck detection method as an input to classical motorway traffic control systems like VSL. The bottleneck probability and the bottleneck length will be used as a reward function for training the reinforcement learning algorithm for controlling the speed limits at VSL signs. Here, the agent will be rewarded if the bottleneck length decreases or the bottleneck probability drops due to a more efficient control strategy.

## Figures and Tables

**Table 1:** Set of fuzzy rules used for bottleneck probability estimation.

	$d_{D}$		$d_{S}$		$p_{b}$
IF	$d_{D}$ is “small”	AND	$d_{S}$ is “small”	THEN	$p_{b}$ is “large”
IF	$d_{D}$ is “small”	AND	$d_{S}$ is “medium”	THEN	$p_{b}$ is “medium”
IF	$d_{D}$ is “small”	AND	$d_{S}$ is “large”	THEN	$p_{b}$ is “small”
IF	$d_{D}$ is “medium”	AND	$d_{S}$ is “small”	THEN	$p_{b}$ is “medium”
IF	$d_{D}$ is “medium”	AND	$d_{S}$ is “medium”	THEN	$p_{b}$ is “medium”
IF	$d_{D}$ is “medium”	AND	$d_{S}$ is “large”	THEN	$p_{b}$ is “small”
IF	$d_{D}$ is “large”	AND	$d_{S}$ is “small”	THEN	$p_{b}$ is “large”
IF	$d_{D}$ is “large”	AND	$d_{S}$ is “medium”	THEN	$p_{b}$ is “medium”
IF	$d_{D}$ is “large”	AND	$d_{S}$ is “large”	THEN	$p_{b}$ is “large”

**Table 2:** Exported data from simulation scenarios.

Parameters	Speed, Density, Location
Collection frequency	1 s
Time interval length	5 min
N. Intervals	24
Simulation time	120 min
N. Vehicle routes	15,541
Motorway length	8000 m
N. Segments (test)	16 (500 m)
N. Segments (validation)	160 (50 m)

**Table 3:** Validation of the threshold for the proposed bottleneck probability estimation method.

Threshold	Class	Precision	Recall	F1-Score	Accuracy
10%	Normal	1.00	0.03	0.06	0.29
	Bottleneck	0.28	1.00	0.44	
20%	Normal	1.00	0.05	0.10	0.31
	Bottleneck	0.28	1.00	0.44	
30%	Normal	1.00	0.06	0.11	0.32
	Bottleneck	0.28	1.00	0.44	
40%	Normal	1.00	0.14	0.24	0.37
	Bottleneck	0.30	1.00	0.46	
50%	Normal	0.94	0.95	0.94	0.92
	Bottleneck	0.86	0.84	0.85	
60%	Normal	0.80	0.99	0.89	0.82
	Bottleneck	0.96	0.36	0.52	
70%	Normal	0.75	1.00	0.86	0.76
	Bottleneck	1.00	0.13	0.23	
80%	Normal	0.75	1.00	0.86	0.76
	Bottleneck	1.00	0.12	0.22	
90%	Normal	0.74	1.00	0.85	0.74
	Bottleneck	1.00	0.05	0.09	

## Data Availability

Data and bottleneck probability method coded in Python programming language are publicly available at https://github.com/tisljaricleo/fuzzy-highway-bottleneck-python (accessed on 19 March 2022).

## Abbreviations

The following abbreviations are used in this manuscript:

CoM	Center of Mass
CAV	Connected Autonomous Vehicle
CV	Connected Vehicle
FIS	Fuzzy Inference System
HCM	Highway Capacity Manual
HDV	Heavy Duty Vehicle
ITS	Intelligent Transport Systems
RM	Ramp Metering
STM	Speed Transition Matrix
SUMO	Simulation of Urban MObillity
VSL	Variable Speed Limit
